# Use of tuf as a target for sequence-based identification of Gram-positive cocci of the genus Enterococcus, Streptococcus, coagulase-negative Staphylococcus, and Lactococcus

**DOI:** 10.1186/1476-0711-11-31

**Published:** 2012-11-27

**Authors:** Xuerui Li, Juan Xing, Baoyu Li, Pu Wang, Jixing Liu

**Affiliations:** 1 State Key Laboratory of Veterinary Etiological Biology, Key Laboratory of Grazing Animal Diseases of the Ministry of Agriculture; Key Laboratory of Veterinary Public Health of the Ministry of Agriculture; Animal Infectious Diseases Research Laboratory, Lanzhou Veterinary Research Institute, CAAS, Lanzhou, 730046, China; 2 The Wistar Institute, Philadelphia, PA, 19104, USA

**Keywords:** *Enterococcus*, *Streptococcus*, *Staphylococcus*, *Lactococcus*, tuf, species identification

## Abstract

**Background:**

Accurate identification of isolates belonging to genus Enterococcus, Streptococcus, coagulase-negative Staphylococcus, and Lactococcus at the species level is necessary to provide a better understanding of their pathogenic potential, to aid in making clinical decisions, and to conduct epidemiologic investigations,especially when large blind samples must be analyzed. It is useful to simultaneously identify species in different genera using a single primer pair.

**Methods:**

We developed a primer pair based on the tuf gene (encoding elongation factor) sequence to identify 56 Gram-positive cocci isolates.

**Results:**

The target sequences were amplified from all 56 samples. The sequencing results and the phylogenetic tree derived from the partial tuf gene sequences identified the isolates as three enterococcal species, two lactococcal species, two staphylococcal species, and six streptococcal species, as well as eight isolates that were novel species of the genus Streptococcus. Partial gene sequence analysis of the sodA, dnaK, and 16S RNA genes confirmed the results obtained by tuf gene sequencing.

**Conclusion:**

Based on the uniform amplification of the tuf gene from all samples and the ability to identify all isolates at both the genus and species levels, we conclude that the primer pair developed in this research provides a powerful tool for identifying these organisms in clinical laboratories where large blind samples are used.

## Background

Bacterial species of the genera Enterococcus, Streptococcus, coagulase-negative Staphylococcus, and Lactococcus are Gram positive cocci in the class Bacilli. Enterococcus, streptococcus, and staphylococcus inhabit a wide range of environments, including the gastrointestinal tracts of humans and animals. Lactococcus is a genus of lactic acid bacteria that were formerly included in the genus Streptococcus, and have been widely used in the production of cheese and milk products.

Accurate identification of isolates belonging to these genus at the species level is necessary to provide a better understanding of their pathogenic potential, to aid in making clinical decisions, and to conduct epidemiologic investigations. Because species identification based on phenotypic characterization is time-consuming and can produce ambiguous results [1-6], molecular identification methods have taken precedence. Of the molecular methods used, polymerase chain reaction (PCR) sequencing-based methods are powerful tools for identifying species both within [7-10] and between genera [11-14]. When large blind samples must be analyzed, it is useful to simultaneously identify species in different genera using a single primer pair. Other than the 16S rRNA gene-based methods [15], several PCR-based sequencing tools have been developed to identify species in the class Bacilli. rpoB gene-based PCR sequencing has been used for accurate detection and identification of species in the genera Streptococcus, Enterococcus, Gemella, Abiotrophia, Granulicatella [11], and Staphylococcus [16]. The groES and groEL genes have also been used as a target for identification of Abiotrophia, Granulicatella, and Gemella species [12].

The tuf gene, which encodes the elongation factor EF-Tu, is involved in peptide chain formation. It is a ubiquitous and highly evolutionarily conserved part of the core genome, and is more discriminative than the 16S rRNA gene for identifying strains belonging to the genera Enterococcus [17], Staphylococcus [18], and Streptococcus [19]. In this study, we developed a simple tuf gene based PCR and sequencing assay to identify isolates belonging to the genera Enterococcus, Staphylococcus, Streptococcus, and Lactococcus. Analysis based on the partial tuf gene sequence revealed that the target could be amplified from all isolates used in this study, and that it is superior to previous techniques for differentiating the strains at the species level.

## Methods

### Bacterial strains

The strains examined in this study included *Streptococcus suis* (S.suis)serotype 2 strain 9801, S. suis 05ZYH33, one Lactococcus lactis subsp. lactis isolate, 48 cocci isolates that was isolated by our lab between 2006 and 2011, and four cocci strains purchased from the Chinese General Microbiology Culture Collection Center (CGMCC), which included the following: *Streptococcus pernyi (S.peryi)* CGMCC1.1010, *Streptococcus salivarius* CGMCC 1.2498, *Streptococcus mutans* CGMCC 1.2499, and *Streptococcus bovis* CGMCC1.2502. *Streptococcus cremoris* (*L. lactis subsp. cremoris*) CICC 20175 was also purchased from the Chinese Center of Industrial Culture Collection (CICC).

### Isolation and sequencing of genomic DNA

Genomic DNA was isolated and purified using a GenElute Bacterial Genomic DNA Kit (Sigma) according to the manufacturer’s instructions. PCR primers complementary to highly conserved regions of the tuf gene were designed ( The primers wer designed on the basis of Streptococcus suis P1/7 tuf gene sequence. However, before designing these, the tuf gene sequences of all the published sequences in genus Streptococcus were aligned to identify sequences that were suitable for primer design. Table 1) and used to amplify partial fragments of the tuf gene from all isolates. The amplification was performed in the personal thermal thermocycler (BioRad MJ-Mini), and PCR conditions were optimized as follows: a total reaction mix of 50 μl contained 0.5 ul (2.5U/ul) of PrimerSTAR HS DNA polymerase (Takara), 5× PrimerSTAR buffer, 4 ul dNTP Mixture (250 μM each), 1 ul of each primer and 1 μl of genomic DNA template. PCR was performed in a DNA thermal cycler with 30 cycles of 94°C for 30 s, 50–55°C for 1 min, 72°C for 1 min 30 s, followed by a final extension step of 72°C for 10 min. The PCR products were collected using Takara MiniBEST DNA Fragment Purification Kit Ver.3.0 and ligated into PMD18 vector (Takara) and three positive clones were sent to the Shanghai Sangon Biotechnology Company for sequencing. The PCR products were sequenced using a BigDye terminator v3.1 cycle sequencing kit (Applied Biosystems) using a PRISM3730 Genetic Analyzer (Applied Biosystems).

**Table 1:** Primers used in this study

**Primers**	**Sequence (5_ to 3_)**	**Gene**	**Primers from**
Tuf-F	5^′^- CCAATGCCACAAACTCGT -3^′^	tuf	This study
Tuf-R	5^′^- CCTGAACCAACAGTACGT -3^′^		
D1	5^′^-CCITAYICITAYGAYGCIYTIGARCC-3^′^	sodA	reference [35,36]
D2	5^′^-ARRTARTAIGCRTGYTCCCAIACRTC-3^′^		
dnaK-F	5^′^CTTGGTGGTGACGACTTTGAC -3^′^	dnaK	This study
dnaK-R	5^′^CCACCCATTGTTTCGATACCA-3^′^		
16s F	5^′^AGAGTTTGATCCTGGCTCAG-3^′^	16S RNA	Reference [37]
16s R	5^′^AAAGGAGGTGATCCAGCC-3^′^		

For the isolates that were determined to be either Staphylococcus or Enterococcus species, partial sodA gene regions were amplified and sequenced to confirm the result, while Streptococcus species isolates were confirmed by amplification and sequencing of the partial dnaK gene. Isolates that could not be identified at the species level following comparison with available tuf gene sequence data published in Genbank and EMBL were further analyzed by 16S rRNA gene amplification and sequence analysis. PCR primers are listed in Table 1

### Phylogenetic relationships

The phylogenetic relationships among species were analyzed using the neighbor-joining method in MEGA 5.0 [20]. For this analysis, distances between the sequences were calculated using Kimura’s two-parameter model [20-23]. Levels of similarity were determined among species. Bootstrap values were obtained for 500 randomly generated trees.

### Nucleotide sequence accession numbers

Nucleotide sequences determined in this study were submitted to GenBank (http://www.ncbi.nlm.nih.gov/genbank/). tuf gene nucleotide sequences were submitted under GenBank accession numbers JX436506–JX436520; sodA nucleotide sequences were submitted under GenBank accession numbers JX436496–JX436497; L. lactis subsp lactis strain LVRI001 dnaK sequence was submitted under GenBank accession number 436495; and 16S rRNA nucleotide sequences were submitted under GenBank accession numbers JQ255459–JQ255462.

## Results

### Identification of isolates

Partial tuf gene sequences were amplified from all 56 strains and then sequenced and compared. In the identified isolates, tuf was 827 bp long in all Staphylococcus warneri strains and 830 bp in all other strains.

NCBI BLASTN analysis revealed that the 48 isolates collected by our lab included eight Streptococcus uberis strains that have 99.8–100% sequence similarity to each other in the tuf gene. The nucleotide sequences of these tuf genes shared approximately 99.8% nucleotide similarity with that of *Streptococcus. uberis* 0140J. Six *Streptococcus thermophilus* (*S. thermophilus*) isolates were identified, all of which showed 100% sequence similarity to the tuf gene of *S. thermophilus* ND03, while a further eight isolates were identified as *Staphylococcus warneri* (*S. warneri*), which showed 100% nucleotide sequence similarity to each other, and 99.5% (777/781 bp) similarity to the tuf gene of *S. warneri* ATCC 27836. Six strains were identified as *Staphylococcus hyicus* (*S. hyicus* )and shared 100% identity with each other and 99.9% (705/706 bp) identity with *S. hyicus* strain CIP 81.58. Seven *Enterococcus faecalis* (*E. faecalis* ) strains were also identified and showed 99.9% identity to the tuf sequence of *E. faecalis* V583. Five *Enterococcus avium* (*E. avium* ) isolates shared 99.6–100% sequence similarity with each other and 98.6% (706/717 bp and 707/717 bp) sequence similarity to *E. avium* ATCC 14025. Finally, we identified eight isolates as belonging to the genus Streptococcus, but could not make a species identification based on comparison with published tuf sequence data in GenBank or EMBL. These sequences shared 99.0–100% sequence similarity with each other, and the highest tuf gene homology was with S. suis A7 (94.8%, CP002570.1 SSUA7_0486 ). The levels of partial tuf gene sequence divergence between the isolates and representatives of other Staphylococcus species were in the range 5.1–14.2%.

The tuf gene diversity was calculated using Kimura’s two-parameter mode using Mega 5.0. At the genus level, the tuf sequence diversity among different species within the genus Streptococcus ranged from 1.1–12.9%. Sequence diversity ranged from 9–12.3% among different species in the genus Enterococcus. For genus Staphylococcus, diversity ranged from 4.7–10.1%.

### Confirmation of results using sodA or dnaK gene sequences

Because the isolates might be misidentified when using a single gene sequence for identification, we used a second partial gene sequence to confirm the result obtained by tuf gene analysis. The sodA gene was used for confirmation of the isolates identified as belonging to the genera Staphylococcus and Enterococcus. For the isolates in the genus Streptococcus, a partial dnaK sequence was amplified and sequenced. The sequencing results confirmed the results obtained from the partial tuf gene analysis.

When *S. warneri* strains used in this research showed 99.5% (777/781 bp) nucleotide sequence (nt) similarity to *S. warneri* ATCC 27836, it also showed 99.5% (767/771) nt sequence similarity to *Staphylococcus pasteuri* (*S. pasteuri* )FI64. For this reason, the partial sodA sequences were then used for analysis. The sodA gene of the *S. warneri* strains isolated in our lab were found to show 99.8% (428/429 bp) sequence similarity to the sodA gene of *S. warneri* CIT S00-147. However, they were clearly distinguished from *S. pasteuri* strain CIP 103540 T, with a nucleotide sequence similarity of only 94.3% (396/420 bp).

### Identification of *S.pernyi* CGMCC1.1010 as *Enterococcus mundtii*

The strain *S. pernyi* CGMCC1.1010 was obtained from the CGMCC. In a recent work by Cui-Fang et al., this strain was suggested to be an enterococcal species [24]. Based on partial tuf gene analysis, we determined that the tuf gene of *S. pernyi* CGMCC1.1010 has 98.9% nucleotide identity (792/801 bp) to that of *E. mundtii* strain ATCC43186. Furthermore, analysis of the partial sodA sequence revealed that sodA of *S. pernyi* CGMCC1.1010 has 99.8% nucleotide identity (437/438 bp) to that of *E. mundtii* strain ATCC 43186. Thus, we confirmed that CGMCC1.1010 is actually an *E. mundtii* strain.

### Phylogenetic analysis of eight Streptococcus isolates

**Figure 1:**
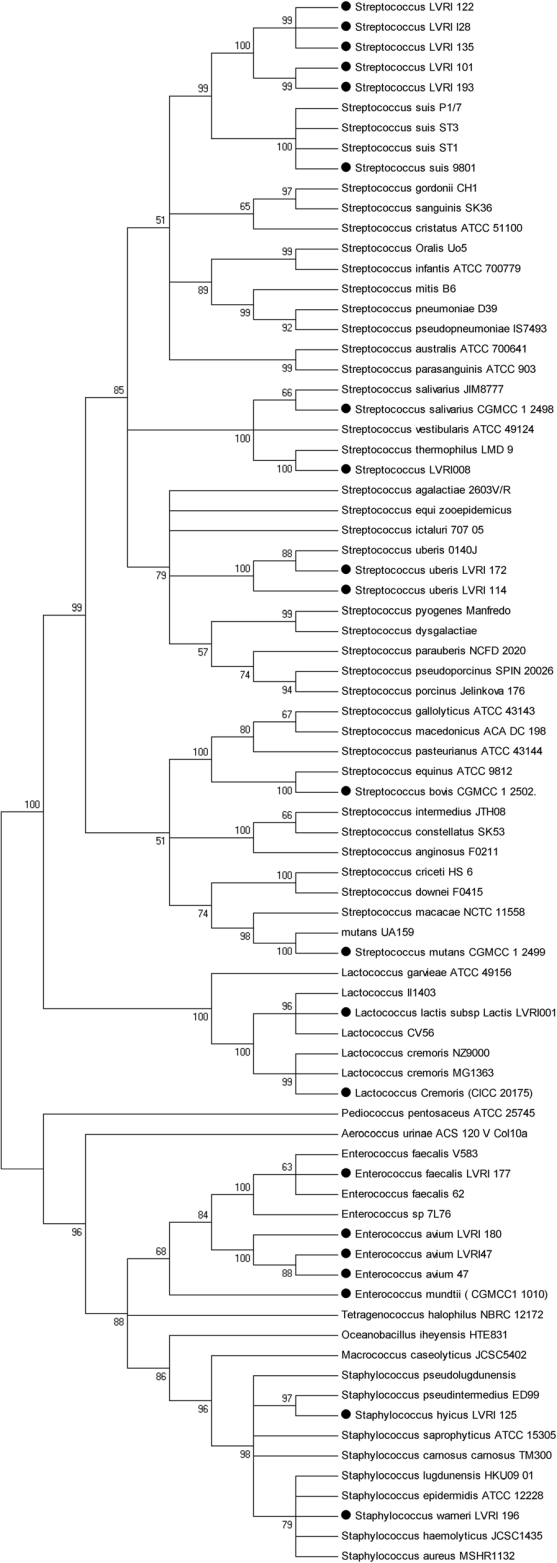
**Phylogenetic tree based on partial *tuf.***

Phylogenetic analysis was carried out using the neighbor-joining method (reference strains and accession numbers are listed in Additional file 1. See Figure 1). Phylogenetic analysis identified 40 of the 48 isolates at both the genus and species levels and agreed with results of the BLAST analysis. The eight isolates that shared 90–100% sequence similarity with each other, but could not be identified at the species level by BLAST analyses were clustered into the same group. They were further analyzed by 16S rRNA gene amplification and sequencing. Preliminary 16S rRNA gene sequence analysis (1550 bp) found that the eight isolates have more than 99.7% similarity to each other. BLAST analyses of these 16S rRNA sequences determined that the isolates showed the greatest sequence similarity to *Streptococcus minor* (*S. minor* )strain LMG 21735 (96.8%, AY232833.1) and *S. minor* strain ON59 (96.8%, AB559734.1). Because isolates that show ≤ 98.7% 16S rRNA sequence similarity are always considered members of a different species [25], we designated these isolates as a novel species of Streptococcus and named this species *Streptococcus parasuis.* Further biochemical research is being conducted to classify and confirm this suspected novel species.

## Discussion

The application of tuf gene analysis to molecular identification has been evaluated for many bacterial species, including Staphylococcus, Streptococcus, Enterococcus, Campylobacter, and Aeromonas species [26,27]. In the current study, we assessed the feasibility of sequencing partial tuf genes for identifying Enterococcus, Staphylococcus, Lactococcus, and Streptococcus species. Sequencing results, a phylogenetic tree derived from the partial tuf gene sequences, and confirmatory sodA, dnaK, and 16S RNA gene sequence analyses revealed that the partial tuf sequence used here could identify the majority of isolates at both the genus and species levels. In addition to identifying isolates of known species, the partial tuf gene sequences could also be used to identify suspected novel species, with a total of eight isolates considered to belong to a novel species, based on tuf and 16S rRNA gene sequence analysis. The data presented here demonstrate that PCR amplification of the tuf gene fragment using our specifically designed primer pair, followed by sequence analysis, is a suitable molecular approach for the identification of Enterococcus, Staphylococcus, Lactococcus, and Streptococcus isolates at the species level.

As it is often difficult to identify isolates at the species level relying only on single gene based primers [28-31], partial gene sequences of sodA, dnaK, or the 16S rRNA gene were also used to confirm our results.

Reliable discrimination between closely related species depends on the variability of the target gene. A high level of variability might be helpful for good discrimination between species, but it can also be a disadvantage because of instability of species-specific signatures and difficulties in developing reliable primers or probes. Because of the high level of variability, the higher the discriminating power, the higher the proportion of strains that are not amplified using a single primer pair [32-34]. In this research, the primer pair was based on *S. suis* sequences. Because the primers were not degenerate, we expected to have to adjust the nucleotide sequences to obtain good amplification from all strains. However, the primer pair worked well and all the appropriate target sequences were amplified from 14 species in four genera (56/56).

The tuf gene-based primer pair designed in the present study may be helpful for the accurate detection and identification of Enterococcus, Staphylococcus, Lactococcus, and Streptococcus species, as well as related genera of medical interest.

## Conclusions

In conclusion, this study confirms that tuf is a good alternative molecular marker for both phylogenetic analysis and species identification of clinical isolates when large blind samples are used. It should be applied to phylogeny as a first-line genomic technique.

## Abbreviations

Tuf: Elongation factor Tu; *S.suis*: *Streptococcus suis*; *S.peryi*: *Streptococcus peryi*; *S. thermophilus*: *Streptococcus thermophilus*; *S. warneri*: *Staphylococcus warneri*; *S. hyicus*: *Staphylococcus hyicus*; *E. faecalis*: *Enterococcus faecalis*; *E. avium*: *Enterococcus avium*; *S. pasteuri*: *Staphylococcus pasteuri*; *E. mundtii*: *Enterococcus mundtii*; *S. minor*: *Streptococcus minor*.

## Competing interests

Authors have no competing interests.

## Authors’ contributions

Xuerui Li, Juanxing and Baoyu Li co-worked on data collection and organisation, performed statistical analysis of the data and contributed to writing and interpretation of the manuscript. Pu wang wrote the manuscript. Jixing Liu contributed to the design and writing of the manuscript. All authors have read and approved the manuscript.

## Supplementary Material

Additional file 1 Sources and gene accession numbers of the bacterial reference strains used in this study. Click here for file
