# Supplementary material for: Use of tuf as a target for sequence-based identification of Gram-positive cocci of the genus Enterococcus, Streptococcus, coagulase-negative Staphylococcus, and Lactococcus
Source: Ann Clin Microbiol Antimicrob. 2012 Nov 27;11:31. doi: 10.1186/1476-0711-11-31 (PMC3533577; doi:10.1186/1476-0711-11-31)
Supplement: Additional file 1 — Sources and gene accession numbers of the bacterial reference strains used in this study. [file 1476-0711-11-31-S1.doc]

**Additional file 1**. Sources and gene accession numbers of the bacterial reference strains used in this study

| Strains | Source | accession number |
| --- | --- | --- |
| Streptococcus | Pneumoniae D39 | Genebank: FQ312027.1 |
| Streptococcus | Pyogenes Manfredo | Genebank: [AM295007.1](http://www.ncbi.nlm.nih.gov/nucleotide/134271134?report=genbank&log$=nucltop&blast_rank=1&RID=UP8K73BK013) |
| Streptococcus | Thermophilus LMD 9 | Genebank: [CP000419.1](http://www.ncbi.nlm.nih.gov/nucleotide/116100249?report=genbank&log$=nucltop&blast_rank=1&RID=UP8T53DZ012) |
| Streptococcus | Salivarius JIM8777 | Genebank: [FR873482.1](http://www.ncbi.nlm.nih.gov/nucleotide/338744031?report=genbank&log$=nucltop&blast_rank=2&RID=UP95NXTZ016) |
| Streptococcus | Agalactiae 2603V/R | Genebank: [AE009948.1](http://www.ncbi.nlm.nih.gov/nucleotide/22535226?report=genbank&log$=nucltop&blast_rank=1&RID=UP9A50VS016) |
| Streptococcus | Equisubsp zooepidemicus ATCC 35246 | Genebank: [CP002904.1](http://www.ncbi.nlm.nih.gov/nucleotide/338846021?report=genbank&log$=nucltop&blast_rank=3&RID=UP9HJ8G001S) |
| Streptococcus | Idemicus H70 | Genebank: [FM204884.1](http://www.ncbi.nlm.nih.gov/nucleotide/225700893?report=genbank&log$=nucltop&blast_rank=1&RID=UP9HUN3D012) |
| Streptococcus | Dysgalactiae subsp equisimilis ATCC 12394 | Genebank: [CP002215.1](http://www.ncbi.nlm.nih.gov/nucleotide/323126259?report=genbank&log$=nucltop&blast_rank=1&RID=UP9SGVAG013) |
| Streptococcus | Gallolyticus subspgallolyticus ATCC 43143 | Genebank: [AP012053.1](http://www.ncbi.nlm.nih.gov/nucleotide/334279325?report=genbank&log$=nucltop&blast_rank=1&RID=UP9YJ8S2012) |
| Streptococcus | Mutans UA159 | Genebank: [AE014133.2](http://www.ncbi.nlm.nih.gov/nucleotide/345287734?report=genbank&log$=nucltop&blast_rank=1&RID=UPA2TUUH016) |
| Streptococcus | Suis P1/7 | Genebank: [AM946016.1](http://www.ncbi.nlm.nih.gov/nucleotide/251819067?report=genbank&log$=nucltop&blast_rank=7&RID=UPA59CES013) |
| Streptococcus | Suis ST3 | Genebank: [CP002633.1](http://www.ncbi.nlm.nih.gov/nucleotide/329306072?report=genbank&log$=nucltop&blast_rank=5&RID=UPA59CES013) |
| Streptococcus | Suis ST1 | Genebank: [CP002651.1](http://www.ncbi.nlm.nih.gov/nucleotide/353739427?report=genbank&log$=nucltop&blast_rank=11&RID=UPA59CES013) |
| Streptococcus | Streptococcus Oralis Uo5 | Genebank: [FR720602.1](http://www.ncbi.nlm.nih.gov/nucleotide/326682110?report=genbank&log$=nucltop&blast_rank=1&RID=UR84HDBN01S) |
| Streptococcus | Mitis B6 | Genebank: [FN568063.1](http://www.ncbi.nlm.nih.gov/nucleotide/288906474?report=genbank&log$=nucltop&blast_rank=1&RID=UR8MJWD501S) |
| Streptococcus | Gordonii str Challis substr CH1 | Genebank: [CP000725.1](http://www.ncbi.nlm.nih.gov/nucleotide/157074445?report=genbank&log$=nucltop&blast_rank=1&RID=UR8RDH5B01S) |
| Streptococcus | Sanguinis SK36 | Genebank: [CP000387.1](http://www.ncbi.nlm.nih.gov/nucleotide/125496804?report=genbank&log$=nucltop&blast_rank=1&RID=UR8VWB7Y01N) |
| Streptococcus | Vestibularis ATCC 49124 | Genebank: [AF276277.1](http://www.ncbi.nlm.nih.gov/nucleotide/14578929?report=genbank&log$=nucltop&blast_rank=5&RID=UR91ZBHD01S) |
| Streptococcus | Infantis ATCC 700779 | Genebank: [EU156917.1](http://www.ncbi.nlm.nih.gov/nucleotide/158262844?report=genbank&log$=nucltop&blast_rank=2&RID=URD1JGT5013) |
| Streptococcus | Pseudopneumoniae IS7493 | Genebank: [CP002925.1](http://www.ncbi.nlm.nih.gov/nucleotide/341932553?report=genbank&log$=nucltop&blast_rank=1&RID=UR996PNC01S) |
| Streptococcus | Pasteurianus ATCC 43144 | Genebank: [AP012054.1](http://www.ncbi.nlm.nih.gov/nucleotide/334281572?report=genbank&log$=nucltop&blast_rank=1&RID=UR9HAJMW01S) |
| Streptococcus | macedonicus ACA DC 198 | Genebank: HE613569.1 |
| Streptococcus | intermedius JTH08 | Genebank: AP010969.1 |
| Lactococcus | CV56 | Genebank: [CP002365.1](http://www.ncbi.nlm.nih.gov/nucleotide/326405597?report=genbank&log$=nucltop&blast_rank=2&RID=UR9MCJAH01N) |
| Lactococcus | Il1403 | Genebank: [AE005176.1](http://www.ncbi.nlm.nih.gov/nucleotide/13400022?report=genbank&log$=nucltop&blast_rank=3&RID=UR9MCJAH01N) |
| Lactococcus | Cremoris NZ9000 | Genebank: [CP002094.1](http://www.ncbi.nlm.nih.gov/nucleotide/300069620?report=genbank&log$=nucltop&blast_rank=5&RID=UR9MCJAH01N) |
| Lactococcus | Cremoris MG1363 | Genebank: [AM406671.1](http://www.ncbi.nlm.nih.gov/nucleotide/124491690?report=genbank&log$=nucltop&blast_rank=6&RID=UR9MCJAH01N) |
| Lactococcus | Garvieae ATCC 49156 | Genebank: [AP009332.1](http://www.ncbi.nlm.nih.gov/nucleotide/343179174?report=genbank&log$=nucltop&blast_rank=2&RID=URD95NNY01N) |
| Lactococcus | Reuteri DSM 20016 | Genebank: [CP000705.1](http://www.ncbi.nlm.nih.gov/nucleotide/148530277?report=genbank&log$=nucltop&blast_rank=2&RID=URCD654W012) |
| Enterococcus | Sp L76 | Genebank: [FP929058.1](http://www.ncbi.nlm.nih.gov/nucleotide/295112306?report=genbank&log$=nucltop&blast_rank=6&RID=UR9WBT0M012) |
| Enterococcus | Faecalis OG1RF | Genebank: [CP002621.1](http://www.ncbi.nlm.nih.gov/nucleotide/327533853?report=genbank&log$=nucltop&blast_rank=7&RID=UR9WBT0M012) |
| Enterococcus | Faecalis V583 | Genebank: [AE016830.1](http://www.ncbi.nlm.nih.gov/nucleotide/29350190?report=genbank&log$=nucltop&blast_rank=8&RID=UR9WBT0M012) |
| Enterococcus | Faecalis 62 | Genebank: [CP002491.1](http://www.ncbi.nlm.nih.gov/nucleotide/323478858?report=genbank&log$=nucltop&blast_rank=9&RID=UR9WBT0M012) |
| Staphylococcus | Pseudintermedius ED99 | Genebank: [CP002478.1](http://www.ncbi.nlm.nih.gov/nucleotide/323463200?report=genbank&log$=nucltop&blast_rank=1&RID=URAAVNU901N) |
| Staphylococcus | Saprophyticus subsp saprophyticus ATCC 15305 | Genebank: [AP008934.1](http://www.ncbi.nlm.nih.gov/nucleotide/72493824?report=genbank&log$=nucltop&blast_rank=1&RID=URAFKNFT01S) |
| Staphylococcus | Carnosus subsp carnosus TM300 | Genebank: [AM295250.1](http://www.ncbi.nlm.nih.gov/nucleotide/222420101?report=genbank&log$=nucltop&blast_rank=1&RID=URAGMF7301S) |
| Staphylococcus | Pseudolugdunensis strain B006 | Genebank: [EF053368.1](http://www.ncbi.nlm.nih.gov/nucleotide/126095416?report=genbank&log$=nucltop&blast_rank=1&RID=URAJPTZB013) |
| Staphylococcus | Haemolyticus JCSC1435 | Genebank: [AP006716.1](http://www.ncbi.nlm.nih.gov/nucleotide/68445725?report=genbank&log$=nucltop&blast_rank=1&RID=URAKE08J01S) |
| Staphylococcus | Aureus 11819_97 | Genebank: [CP003194.1](http://www.ncbi.nlm.nih.gov/nucleotide/364521287?report=genbank&log$=nucltop&blast_rank=3&RID=URANMWW801S) |
| Staphylococcus | Aureus MSHR1132 | Genebank: [FR821777.2](http://www.ncbi.nlm.nih.gov/nucleotide/356871517?report=genbank&log$=nucltop&blast_rank=1&RID=URASUV83016) |
| Staphylococcus | Epidermidis ATCC 12228 | Genebank: [AE015929.1](http://www.ncbi.nlm.nih.gov/nucleotide/27316888?report=genbank&log$=nucltop&blast_rank=2&RID=URATNXMA01N) |
| Staphylococcus | Lugdunensis HKU09 01 | Genebank: [CP001837.1](http://www.ncbi.nlm.nih.gov/nucleotide/289178903?report=genbank&log$=nucltop&blast_rank=2&RID=URAW27DP01S) |
| Macrococcus | Caseolyticus JCSC5402 | Genebank: [AP009484.1](http://www.ncbi.nlm.nih.gov/nucleotide/222119372?report=genbank&log$=nucltop&blast_rank=1&RID=URAZXPXW01S) |
| Tetragenococcus | Halophilus NBRC 12172 | Genebank: [AP012046.1](http://www.ncbi.nlm.nih.gov/nucleotide/348600280?report=genbank&log$=nucltop&blast_rank=1&RID=URB1XB8M013) |
| Oceanobacillus | Iheyensis HTE831 | Genebank: [BA000028.3](http://www.ncbi.nlm.nih.gov/nucleotide/42632302?report=genbank&log$=nucltop&blast_rank=1&RID=URB8W1XH016) |
| Aerococcus | Urinae ACS 120 V Col10a | Genebank: [CP002512.1](http://www.ncbi.nlm.nih.gov/nucleotide/326650014?report=genbank&log$=nucltop&blast_rank=1&RID=URB5TAYZ013) |
| Pediococcus | Pentosaceus ATCC 25745 | Genebank: [CP000422.1](http://www.ncbi.nlm.nih.gov/nucleotide/116101968?report=genbank&log$=nucltop&blast_rank=1&RID=URBCY0E5016) |
| Streptococcus | Equinus ATCC 9812 | EMBL AEVB01000027 |
| Streptococcus | Cristatus ATCC 51100 | EMBL AEVC01000007 |
| Streptococcus | Australis ATCC 700641 | EMBL AEQR01000019 |
| Streptococcus | Anginosus F0211 | EMBL AECT01000035 |
| Streptococcus | Parauberis NCFD 2020 | EMBL AEUT02000001 |
| Streptococcus | Pseudoporcinus SPIN 20026 | EMBL AENS01000013 |
| Streptococcus | Parasanguinis ATCC 903 | EMBL AEVE01000032 |
| Streptococcus | porcinus_str Jelinkova 176 | EMBL AEUU02000001 |
| Streptococcus | Downei F0415 | EMBL AEKN01000003.1 |
| Streptococcus | Criceti HS 6 | EMBL AEUV02000002.1 |
| Streptococcus | Ictaluri 707 05 | EMBL AEUX02000005.1 |
| Streptococcus | Macacae NCTC 11558 | EMBL AEUW02000001.1 |
| Streptococcus | Constellatus subsp constellatus SK53 | EMBL AICQ01000008.1 |
